# Contraindicated drug–drug interactions and associated adverse drug reactions in an observational cohort study of 4543 paediatric hospitalized patients

**DOI:** 10.1002/bcp.70557

**Published:** 2026-04-28

**Authors:** Emilie Laval, Sylvain Goutelle, Inesse Boussaha, Xavier Dode, Yanis Mimouni, Kim An Nguyen, Behrouz Kassai

**Affiliations:** ^1^ Department of Pharmacotoxicology Hospices Civils de Lyon, CIC 1407 de Lyon, INSERM, CHU‐Lyon Bron France; ^2^ UMR CNRS 5558, Laboratoire de Biométrie et Biologie Humaine Equipe Evaluation et Modélisation des Effets Thérapeutiques Lyon France; ^3^ Department of Pharmacy Hospices Civils de Lyon, CHU‐Lyon Bron France; ^4^ Thematic Institute of Genetics, Genomics & Bioinformatics, INSERM Paris France

**Keywords:** adverse drug reactions, drug interactions, medication safety, paediatrics, pharmacoepidemiology, pharmacovigilance

## Abstract

**Background and Purpose:**

Drug–drug interactions (DDIs) are associated with an increased risk of adverse drug reactions (ADRs). Hospitalized children are particularly vulnerable to DDIs and ADRs due to polypharmacy, frequent use of unlicensed or off‐label medications, and dosing regimens often extrapolated from adult data. The aims of this study were to characterize contraindicated DDIs detected in paediatric hospital wards and to assess whether these interactions were associated with ADRs.

**Experimental Approach:**

Medication and ADR data were prospectively collected over a 2‐year observational period in hospitalized children. Patients aged ≤15 years, hospitalized for >3 days and receiving at least two medication during their stay were eligible. Potential contraindicated DDIs were identified using the algorithm implemented in the Thériaque database, based on the official DDI Thesaurus issued by the French National Agency for the Safety of Medicines and Health Products (ANSM).

**Key Results:**

A total of 4543 hospitalized children were included; 12.5% experienced at least one DDI, and 4.3% had at least one contraindicated DDI. Overall, 4225 DDIs were identified, including 709 classified as contraindicated. Thus 72% of the contraindicated DDIs occurred in three departments: paediatric cardiology, developmental psychopathology ward and pulmonology/allergology/cystic fibrosis. Most contraindicated DDIs (80%) were observed in children aged 2–11 years and 12–15 years.

**Conclusion and Implications:**

Although a large number of DDIs were detected in hospitalized paediatric patients, contraindicated DDIs were rarely associated with clinically relevant ADRs. These findings highlight the need to improve the specificity and clinical relevance of DDI alert systems in paediatric inpatient settings.

**What is already known about this subject**

- DDIs and ADRs are a global public health problem.
- Hospitalized children are vulnerable to DDIs and ADRs.

**What this study adds**

- A total of 12.5% of patients had a prescription with at least one DDI.
- A total of 4.3% of patients had a prescription with at least one DDI classified as contraindicated.
- A large number of DDIs were detected, but very few had clinical consequences.

## 1 INTRODUCTION

A drug–drug interaction (DDI) is defined as a modification in the pharmacological or clinical response to a drug when it is administered concomitantly with another drug, based on the known effects of each agent.^1^, ^2^ Identification of DDIs is essential, as they may lead either to reduced therapeutic efficacy or, conversely, to increased drug exposure or pharmacodynamic activity, thereby elevating the risk of adverse drug reactions (ADRs). DDIs that do not result in clinically or biologically significant effects for the patient are referred to as potential DDIs.

Some DDIs are formally classified as contraindicated because of the serious outcomes they may cause, including death, life‐threatening conditions, hospitalization, or prolonged hospital stay. Such contraindications are considered absolute and must not be overridden, as the risk to the patient outweighs any potential benefit. In these circumstances, alternative therapeutic options within the same pharmacological class or from a different class should be prescribed to avoid exposure to a high‐risk interaction.

DDIs and ADRs represent a major public health concern and are associated with increased rates of hospitalization, longer hospital stays, higher healthcare costs, and increased mortality.^3^, ^4^ In the United States, DDIs are estimated to account for between 2600 and 220 000 emergency department visits annually and affect approximately 1.9 to 5 million hospitalized patients.^5^, ^6^ ADRs have been reported as the fourth to sixth leading cause of death among hospitalized patients.^7^ The World Health Organization estimates that at least 60% of ADRs are preventable, particularly those related to DDIs.^8^, ^9^

In hospitalized paediatric populations, the prevalence of DDIs ranges from 49% to 75%,^10^, ^11^, ^12^, ^13^ while the reported incidence of ADRs varies widely, from 0.6% to 42%.^14^, ^15^, ^16^, ^17^ Polypharmacy is highly prevalent among hospitalized children, particularly those with prolonged hospital stays or rare diseases.^18^ Exposure to multiple concomitant medications increases the likelihood of DDIs and, consequently, the risk of ADRs.^10^, ^19^

Several factors predispose children to ADRs, including young age, polypharmacy,^20^ and the frequent use of medications for unlicensed indications or off‐label use in paediatric populations.^10^, ^19^, ^21^, ^22^, ^23^ Children may respond differently to drug therapy than adults due to developmental differences in drug absorption, distribution, metabolism, and excretion. The maturation of hepatic and renal systems is incomplete, particularly in neonates and infants, resulting in reduced expression or absence of cytochrome P450 enzymes, decreased renal blood flow, glomerular filtration, and tubular function. Consequently, drug clearance is reduced and elimination half‐life is prolonged. In addition, paediatric dosing is often extrapolated from adult data and adjusted for body weight, despite important pharmacokinetic differences beyond body size, such as variations in cellular permeability and the ratio of total body water to fat‐free mass.^24^, ^25^

The increasing number of medications prescribed per patient makes the risk of DDIs a daily challenge in routine hospital practice. However, additional data are required to accurately quantify the burden of DDIs and to better understand their contribution to ADRs in paediatric populations.^26^, ^27^

The objectives of this study are (a) to estimate the prevalence and describe the characteristics of potential contraindicated DDIs in hospitalized children and (b) to assess the clinical impact of contraindicated DDIs in this population.

## 2 METHODS

### 2.1 Study design and setting

This descriptive analysis was based on data collected over a 2‐year period (September 2013 to September 2015) in 21 paediatric wards of teaching hospitals in Lyon, France, within the Hôpital Femme Mère Enfant (HFME). HFME is a tertiary care hospital entirely dedicated to women, mothers, couples, and children, providing multidisciplinary care across all paediatric medical and surgical specialties, as well as comprehensive pregnancy‐related care.

HFME combines primary, secondary, and tertiary paediatric services and is among the largest paediatric hospitals in Europe. It comprises 308 paediatric beds (37 869 hospital stays, 82 276 emergency department visits, and 139 911 outpatient consultations in 2019) and 44 neonatology beds (714 hospital stays in 2019).

This study was conducted in accordance with the Strengthening the Reporting of Observational Studies in Epidemiology guidelines for observational research.

### 2.2 Ethics statement

Patient consent was waived because this was a non‐interventional study based exclusively on routinely collected clinical data. All data were coded using nontraceable anonymized identifiers to ensure confidentiality of paediatric patient information.

The data analysed originated from the EREMI database. The EREMI study was conducted in accordance with the Declaration of Helsinki and received approval from the French National Data Protection Authority (Commission nationale de l'informatique et des libertés, CNIL; Ref. MMS/MTE/AR1411279), the Advisory Committee on Information Processing in Health Research (Comité Consultatif sur le traitement de l'information en matière de recherche dans le domaine de la santé, CCTIRS; Ref. 13.546), and the regional ethics committee (Comité d' Ethique des Centres d' Investigation Clinique [CECIC], Rhône‐Alpes‐Auvergne, Clermont‐Ferrand; IRB approval number: IRB5891; authorisation obtained on 20 July 2016), in compliance with the European General Data Protection Regulation (GDPR). The study was registered at ClinicalTrials.gov under the identifier NCT02852590.

### 2.3 Inclusion and exclusion criteria

Inclusion criteria were as follows: (a) patients aged 0 to 15 years, (b) hospital stays of at least 3 days, and (c) treatment with more than one drug during hospitalization.

Patients aged 15 to 18 years were excluded because mortality in this age group is predominantly related to accidents, violence, or suicide rather than diseases requiring hospitalization. Patients hospitalized for an ADR, in the context of voluntary drug intoxication, or without any drug prescription during hospitalization were also excluded.

Prescriptions for blood‐derived products, contrast agents, oxygen or other medical gases, investigational drugs used in clinical research, and nonmedicated topical products (e.g.. moisturizers, ointments, lotions) were not considered in the EREMI study. Procedural practices were not excluded.

### 2.4 Data sources

All analysed data were extracted from the EREMI study database. EREMI is an observational, prospective, multicentre pharmacoepidemiological cohort study designed to evaluate the association between unlicensed or off‐label drug use and ADRs in hospitalized children in France. The detailed EREMI study protocol has been previously published.^28^

The EREMI database includes comprehensive information on all administered drugs as well as patient demographic characteristics, including age, sex, and diagnosis.

### 2.5 Data management

#### 2.5.1 DDIs identification and classification

In this study, all identified DDIs were considered *potential* DDIs. DDIs were detected from drug prescriptions using an interface with the Thériaque database, a drug information system widely used in French hospitals and approved by the French National Authority for Health since 2009. DDI information in Thériaque is based on French summaries of product characteristics and the official DDI Thesaurus issued by the French National Agency for the Safety of Medicines (ANSM).

Prior to its use in the EREMI project, the Thériaque algorithm was evaluated in a pilot cohort of 100 patients through comparison with manual investigator assessments. The analysis showed a high level of concordance, with a Cohen's kappa coefficient of 0.95.

The sequence of drug administration was not incorporated into the study methodology. Potential DDIs were assessed by the Thériaque algorithm, without taking into account the patient's age, over a predefined 24‐h window by identifying all medications administered to a given patient that could theoretically interact with any other medication administered during the same period. Although a drug is generally considered to have negligible pharmacological activity after approximately five to seven elimination half‐lives, a more precise assessment would have required individual evaluation of each prescribed medication to determine, based on its half‐life, whether drug A remained present at the time of administration of drug B. Such an approach would have needed to be applied to all medications for each patient. Given that drug half‐life is influenced by multiple factors and varies substantially between individuals, incorporating these parameters was not feasible in a cohort of 4543 patients. Therefore, a 24‐hour assessment window was chosen as a pragmatic approach to optimize the detection of potential drug–drug interactions across the study population.

DDI detection was performed retrospectively after completion of the study period in 2015.

Based on the Thériaque database, which contains a list of DDIs and their severity classifications, the algorithm identified DDIs according to the two highest levels of seriousness defined by the ANSM:

- **Contraindicated**: ‘This interaction has an absolute character that must not be violated. In practice, it is imperative to prescribe another drug of the same class (or another class) that does not expose the patient to this risk of interaction’.
- **Combination not recommended**: ‘This combination should most often be avoided, except after a thorough evaluation of the benefit–risk ratio. The consequences are certain (sometimes serious) but, in some clinical circumstances, unavoidable. Close patient monitoring is required’.

Only contraindicated DDIs were analysed further. This choice was based on legal considerations, as contraindicated drug combinations are prohibited under French public health law. All DDIs classified as contraindicated by the algorithm were manually reviewed by the authors to confirm their status according to the ANSM Thesaurus.

Notably, when two drugs contained the same active substance, the Thériaque algorithm could detect DDIs if the route of administration or dosage differed. In addition, when a formulation did not allow the full prescribed dose to be administered at once, the algorithm could erroneously identify DDIs in cases where the same drug was administered simultaneously via the same route at different dosages to achieve the prescribed dose.

Only contraindicated DDIs were further analysed. The choice to study only contraindicated DDIs is based on a legal reason because it is prohibited by the public health code. All DDIs detected and classified as contraindicated by the algorithm were manually checked by the authors to determine whether they were indeed contraindicated according to the ANSM's Thesaurus.

Notably, if two drugs had the same active molecule, Theriaque algorithm detected DDIs when the route of administration or the dosage differed. When, the drug formulation did not allow the full dose to be administered at once, the algorithm may wrongly detect DDIs in cases where the same drug was administered simultaneously via the same route of administration but at different dosages to reach the prescribed dose.

For some DDIs, the contraindication applied only under specific conditions (Table 1). For example, erythromycin was associated with a risk of QT interval prolongation only when administered intravenously, while interactions involving lysine acetylsalicylate were considered contraindicated only at high dosages. These conditions were systematically verified through review of patients' medical records and detailed information on administered drugs.

**TABLE 1:** Verification of the criteria required to validate the contraindicated level of certain DDIs.

Criteria required to validate the contraindicated	Description of the verification performed. Reasons for non‐verification, if applicable	Name of the medications involved in the drug interaction	Number of verified DDIs whose classification as contraindicated is validated	Number of verified DDIs that should not have been classified as contraindicated after verification	Number of drug interactions that could not be verified
Contraindicated with IV calcium salts unless supplemented with parenteral nutrition	Calcium salts was administered intravenously at each administration. There was no potassium supplementation in connection with the administration of calcium salts.	Digoxin/potassium (Polyionique B®)	2	0	0
		Subtotal	2	0	0
Contraindicated if Erythromycin is administered intravenously	Erythromycin was administered intravenously each time.	Erythromycin/cyamemazine Erythromycin/domperidone Erythromycin/Escitalopram Erythromycine/hydroxyzine	14 1 3 10	0 0 0 0	0 0 0 0
		Subtotal	28	0	0
Contraindicated unless hypokalemia exists	Potassium levels were checked for hypokalemia before medication administration, when this data was available.	Potassium canreonate + Trometamol/Potassium (CHL®) Potassium canreonate + trometamol/potassium (Numetah®) Potassium canreonate + trometamol/potassium (Glucidion®) Spironolactone/potassium (Numetah®) Spironolactone/potassium (CHL®) Spironolactone/potassium (GLUCO®) Spironolactone/potassium (Diffu‐K®) Spironolactone/potassium (Glucidion®) Spironolactone/potassium canreonate + Trometamol Spironolactone/spironolactone Spironolactone/potassium (Transipeg®) Spironolactone/potassium (RCA®)	34 25 19 10 26 19 2 35 9 0 2 3	17 1 1 0 5 1 3 2 0 0 0 1	7 0 0 0 2 3 2 5 0 1 0 1
		Subtotal	184	31	21
Contraindication in case of concomitant administration	The time of administration of the medications was checked to determine whether they were administered concurrently or not, when this data was available.	Amikacine/amikacine Amikacine/gentamicin Amikacine/tobramycine base Gentamicin/gentamicin Tobramycine sulfate/tobramycine base Tobramycine sulfate/tobramycine sulfate	60 0 0 28 0 68	1 4 2 3 2 0	0 0 0 1 0 0
		Subtotal	156	12	1
Contraindication for doses: ≥1 g per dose and/or ≥3 g per day OR ≥500 mg per dose and/or <3 g per day and in case of history of peptic ulcer	The amount of lysine acetylsalicylate administered per 24 h was verified and never meet the criteria for a contraindication.	Lysine acetylsalicylate/fluindione Lysine acetylsalicylate/warfarin	0 0	31 2	0 0
		Subtotal	0	33	0
		Total	370	76	22

#### 2.5.2 Identification and evaluation of ADRs

ADRs were identified using two complementary approaches: spontaneous reporting by physicians or nurses during routine clinical practice, and active detection by clinical research teams through review of electronic health records using trigger tools.

A trigger is defined as a data element within a health record that signals the occurrence or imminent occurrence of an ADR. Trigger tools consist of targeted medical record reviews performed either manually or automatically. In total, 36 triggers were selected for this study, classified into three categories (clinical, biological, and medication‐related triggers), each with a positive predictive value of at least 10%.^29^

All suspected ADRs were transmitted to the Regional Pharmacovigilance Centre (RPC) for evaluation. The RPC assessed the nature, seriousness, and outcome of ADRs, as well as the suspected drugs, according to World Health Organization criteria. Causality was evaluated using both the French method^30^ and the Naranjo algorithm.^31^

All ADRs, including those not retained or confirmed by the RPC, were subsequently reassessed by an Adjudication Committee (AC). The AC comprised experts: four clinician–professors in paediatrics, one paediatrician–pharmacologist, one hospital pharmacist, and two pharmacovigilance specialists. The AC reassessed ADRs using the same criteria as the RPC, with the exception of the French causality assessment method.

The detailed protocol for ADR evaluation within the EREMI study by both the RPC and the AC has been published previously.^32^

#### 2.5.3 Assessment of the biological impact of contraindicated DDIs detected by the Theriaque algorithm

To assess the potential clinical impact of contraindicated DDIs on patient health, an additional analysis was performed beyond ADR detection using trigger tools. This analysis evaluated whether the identified DDI risk was associated with changes in relevant biological parameters or the occurrence of a related clinical event during hospitalization.

Specifically, biological abnormalities were assessed according to the expected risk with the DDI, including an international normalized ratio (INR) above the therapeutic target for DDIs associated with haemorrhagic risk, elevated serum potassium levels for DDIs associated with hyperkalaemia, and increased serum creatinine levels for DDIs associated with renal impairment. The occurrence of associated clinical events potentially attributable to the DDI during hospital stay was also investigated.

### 2.6 Data and statistical analysis

Patients were classified into four age categories according to ICH E11 guidelines^33^: newborns (0–27 days), infants (1–23 months), children (2–11 years), and adolescents (12–15 years).

Demographic and clinical characteristics at inclusion were described using means, medians, standard deviations, and interquartile ranges for quantitative variables, and frequencies and percentages for qualitative variables.

DDIs were analysed on a daily basis according to drug administration and international nonproprietary names, except for drugs listed in Table 2.

**TABLE 2:** Names given to potassium‐containing drugs for this analysis.

Trade name of drug	Name used for this analysis
Potassium CHL®	Potassium (CHL®)
Diffu‐K ®	Potassium (Diffu‐K®)
Glucidion ®	Potassium (Glucidion®)
Potassium RCA ®	Potassium (RCA®)
Numetah®	Potassium (Numetah®)
Polyionique B®	Potassium (Polyionique®)
Potassium Gluco ®	Potassium (Gluco®)
Transipeg®	Potassium (Transipeg®)

All analyses were conducted on data extracted in 2019. Descriptive analyses were performed using Microsoft® Excel® for Microsoft 365 MSO, and statistical analyses were conducted using RStudio software (version 4.4.2). A two‐sided *p* value <.05 was considered statistically significant for all comparisons.

Comparisons of median age between patients without contraindicated DDIs and those with at least one contraindicated DDI were performed using the Mood's median test. Comparisons of hospital length of stay between these two groups were conducted using a Welch two‐sample *t* test. Prior to this analysis, equality of variances was assessed using an *F* test. Sample sizes were sufficiently large (≥30 per group).

Differences in the frequency of hospitalisations with at least one contraindicated DDI between departments were analysed using Pearson's chi‐squared test. As the expected frequencies were greater than five, the orthopaedic, traumatology, and plastic surgery ward and the emergency ward were grouped for this analysis.

### 2.7 Nomenclature of targets and ligands

Key protein targets and ligands in this article are hyperlinked to corresponding entries in https://www.guidetopharmacology.org and are permanently archived in the Concise Guide to PHARMACOLOGY 2023/24.^34^, ^35^, ^36^

## 3 RESULTS

### 3.1 The characteristics of the study population

Over a two‐years period, data from 4543 patients hospitalized in 21 paediatric wards in Lyon hospital were analysed. Among them, 195 of 4543 patients (4.3%) experienced at least one contraindicated DDI during their hospitalization.

A total of 4225 DDIs were identified, of which 709 were contraindicated. There were 172 unique DDI pairs, including 47 unique contraindicated DDI pairs.

Patient characteristics are presented in Table 3.

**TABLE 3:** Patient characteristics.

Characteristic	EREMI population			EREMI population without contraindicated DDI			EREMI population with at least one contraindicated DDI		
	Total	Male	Female	Total	Male	Female	Total	Male	Female
**Gender—*n* (%)**									
Newborns	584 (12.9)	360	224	575 (13.2)	352	223	9 (4.6)	8	1
Infants	1726 (38.0)	978	748	1690 (38.9)	958	732	36 (18.5)	20	16
Children	1577 (34.7)	846	731	1490 (34.3)	802	688	87 (44.6)	44	43
Adolescents	656 (14.4)	313	343	593 (13.6)	280	313	63 (32.3)	33	30
All age groups combined	4543 (100.0)	2497 (54.9)	2046 (45.1)	4348 (100.0)	2392 (55.0)	1956 (45.0)	195 (100.0)	105 (53.8)	90 (46.2)
**Age—median (IQR), days**									
Newborns	15 (9–20)			15 (9–20)			8 (2–13)		
Infants	136.5 (62–307)			136 (62–307)			182 (86–271.8)		
Children	2345 (1419–3447)			2303.5 (1398.5–3436.5)			2768 (1721–3632.5)		
Adolescent	5001.5 (4731–5243)			4991 (4725–5230)			5098 (4825–5275.5)		
All age groups combined	662 (76–3241.5)			588 (73–3109.8)			3196 (1117.5–4785.5)		
Statistical comparison of median age between patients without contraindicated DDI and those with at least one contraindicated DDI				Mood test *Z* = 7.6883, *p* value = 1.488e−14					
Number of hospitalizations during the study period	6110			5903			207		
Statistical comparison of hospital stays between patients without contraindicated DDI and those with at least one contraindicated DDI				*F* test to compare two variances *F* = 0.067266, *p* value <2.2e−16 Welch two‐sample *t* test *t* = −11.147, *p* value <2.2e−16					
Number of hospitalizations per patient—median (IQR)				1 (1–1)			1 (1–1)		
Number of CI DDI per patient—median (IQR)							2 (1–4)		

Abbreviations: DDI, drug–drug interaction; IQR, interquartile range.

The primary diagnoses of the patients are detailed in Table 4. The primary diagnosis corresponds to the health condition that prompted the patient's admission to the hospital unit. It is determined at discharge. According to this definition, a condition that was not present at admission but occurred during the hospital stay cannot be considered the primary diagnosis. The primary diagnosis was determined in accordance with the clinical situation guidelines and with consideration of the coding options provided by the 10th revision of the International Classification of Diseases (ICD‐10).

**TABLE 4:** Primary diagnoses by hospital stay with at least one contraindicated DDI according to the 10th revision of the International Classification of Diseases (ICD‐10).

ICD‐10	Newborns	Infants	Children	Adolescents	All age groups combined	
	*n*	*n*	*n*	*n*	*n*	%
Unknown	3	18	32	16	69	33.5
Q00‐Q99: Congenital malformations	6	6	17	11	40	19.3
F00‐F99: Mental and Behavioral Disorders		27	6		33	15.9
J00‐J99: Disorder of Respiratory System		4	6	2	12	5.8
N00‐N99: Disorders of Genitourinary System		1	9		10	4.8
S00‐T98: Trauma, Poisoning and external causes		3	4		7	3.4
I00‐I99: Disorder of Circulatory System		2	2	2	6	2.9
K00‐K93: Disorder of Digestive System		1	5		6	2.9
M00‐M99: Disorder of Musculoskeletal System		2	3	1	6	2.9
E00‐E99: Endocrine, Nutritional and Metabolic Disorder		1	1	2	4	1.9
R00‐R99: Not classified			3	1	4	1.9
K00‐K93: Disorder of Digestive System		1	3		4	1.9
G00‐G99: Disorder of Nervous System		1	1	1	3	1.4
C00‐D48: Neoplasms			2		2	1.0
A00‐B99: Infectious and Parasitic Disorder			1		1	0.5
Total	9	67	95	36	207	100

*Note*: The primary diagnosis corresponds to the health condition that prompted the patient's admission to the hospital unit. The primary diagnosis was determined in accordance with the clinical situation guidelines and with consideration of the coding options provided by the ICD‐10.

The median age is differed significantly between patients with at least one contraindicated DDI and those without a contraindicated DDI (*p* < .05) (Table 3).

The median age was 19 months in patients without contraindicated DDI and 8.8 years in those with at least one contraindicated DDI. The presence of contraindicated DDIs was not associated with an increased number of hospital stays (*p* < .05) (Table 3).

In addition, the proportion of patients with at least one contraindicated DDI increased with age groups: 1.5% (9/584) in newborns, 2.1% (36/1726) in infants, 5.5% (87/1577) in children, and 9.6% (63/656) in adolescents.

In the EREMI population, 10.9% (664/6110) of hospital stays included at least one DDI, while 3.4% (*N* = 207/6110) included a least one contraindicated DDI.

Among the population with at least one DDI, 31.2% (*N* = 207/664) of hospital stays were with at least one contraindicated DDI: 22% (9/41) for newborns' hospital stay, 23.4% (36/154) for infants, 29.1% (95/325) for children, and 46.5% (67/144) for adolescents.

The percentage of hospitalisations with at least one contraindicated DDI by department is presented in Table 5. The frequency of the number of hospitalisations with at least one contraindicated DDI is significantly different between departments (*p* < .05) (Table 5).

**TABLE 5:** Percentage of hospital stays with at least one contraindicated drug–drug interaction.

Department	Number of hospital stays for patient who had at least one contraindicated DDI during their hospital stay					Percentage of hospital stay with at least one contraindicated DDI per department (%)
	Newborns (*n*)	Infants (*n*)	Children (*n*)	Adolescents (*n*)	All age groups combined (*n*)	
Developmental psychopathology ward			8	39	47	10.4 (47/451)
Cardiology ward	9	27	39	9	84	7.6 (84/1112)
Orthopaedic, traumatological and plasty surgery ward, emergency ward			4 1	3 0	7 1	6.1 (8/131)
Hepato‐gastrology ward		4	7	2	13	2.9 (13/453)
Nephrology and rheumatology ward		3	11	2	16	2.6 (16/604)
Metabolic and hereditary diseases ward						2.3 (4/172)
Pulmonology, allergology and cystic fibrosis ward			13	6	19	2.0 (19/932)
Urogenital, visceral and thoracic surgery ward			3		3	1.5 (3/204)
Neurology ward			3	3	6	0.8 (6/714)
Endocrinology and diabetology ward		1	4	2	7	0.5 (7/1494)
Total	9	36	95	67	207	
Statistical test comparing the frequency of the number of hospitalizations with at least one contraindicated DDI across departments	Pearson's chi‐squared test *χ* ^2^ = 200.28, *p* value <2.2e−16					

Abbreviation: DDI, drug–drug interaction.

### 3.2 Verification of criteria required to validate the contraindicated level of certain DDIs – Table 1

Among the 709 DDIs classified as contraindicated by the Theriaque algorithm, 468 were conditional, depending on specific criteria like intravenous calcium salts unless supplemented with parenteral nutrition, contraindicated if erythromycin is administered intravenously, contraindicated unless hypokalaemia exists, contraindication in case of concomitant administration of two aminoglycosides, contraindication for doses of lysin acetylsalicylate: ≥1 g per dose and/or ≥3 g per day or ≥500 mg per dose and/or <3 g per day and in case of history of peptic ulcer. These defined criteria were not verified by the algorithm; 446 DDIs could be verified by consulting the medical record (Table 1).

Among them, 370 DDIs were validated as contraindicated and 76 should not have been classified as contraindicated DDIs after verification. The absence of serum potassium values in the patient's medical record is the main cause of the non‐verification of criteria that could not be verified.

### 3.3 Characteristics of DDI

Overall, 72% of the contraindicated DDIs occurred in only three departments: developmental psychopathology, cardiology and pulmonology.

Forty two per cent (297/709) of all contraindicated DDIs were observed in the paediatric cardiology department. Contraindicated DDIs occurred predominantly in the children group (56%, 167/297) in this ward. Potassium‐sparing diuretics particularly aldosterone antagonists, were involved in 67% (199/297) of these DDIs. In 60% (177/297) of cases, these drugs were associated with blood substitutes and IV fluids containing electrolytes. The primary risk was hyperkalaemia by combination of two agents increasing potassium serum level.

Examples of such DDI were potassium canrenoate – trometamol + potassium (CHL®) 18% (52/297), spironolactone + potassium (Glucidion®) 12% (36/297), and spironolactone + potassium (CHL®) 11% (33/297).

The fourth most frequent DDI was the association of lysine acetylsalicylate with a vitamin K antagonist (fluindione) (31/297). The risk associated with this DDI is an increased risk of bleeding.

In the developmental psychopathology ward, 121 were observed, primarily in adolescents (83%, 101/121).

The majority of patients were hospitalized for behavioural and emotional disorders with onset usually occurring in childhood and adolescence. Psycholeptic or psychoanaleptic agents were involved in all DDIs and most cases (93%, 113/121) were DDI between the same class of agents. The DDI between cyamemazine and hydroxyzine was reported in 70% (85/121) of cases, which increased the risk of ventricular arrhythmias by addition of the effects of the two QT‐prolonging drugs.

In the pulmonology, allergology and cystic fibrosis ward, 92 DDI were reported, mainly in children (84%, 77/92).

The majority of patients were hospitalized for bacterial pneumonia.

Aminoglycoside agents (tobramycin, amikacin or gentamicin) were involved in 86% (79/92) of cases. The most frequent DDI detected was the association of ≥2 tobramycin presentations (76%, 70/92). The putative risk associated with this DDI was an increased risk of nephrotoxicity and ototoxicity.

### 3.4 Biological and clinical consequences of DDI

Only one ADR involving QT interval prolongation occurred, in a patient with cyamemazine/hydroxyzine.

No clinical or biological consequences were observed.

Table 6 shows the biological and clinical impact of contraindicated DDIs:

- Hyperkalaemia occurred in 21% (50/236) of contraindicated DDIs between the drugs potassium canrenoate + trometamol with potassium (CHL®, Glucidion®, Numetah®) or spironolactone, spironolactone with potassium (CHL®, Diffu‐K®, Glucidion®, Gluco®, RCA®) or spironolactone.
- Serum creatinine over the normal range occurred in 9% (15/169) of contraindicated DDI. The associated DDI were combination of different presentation of the same aminoglycoside drug, as described above.
- International Normalized Ratio (INR) values higher than the target INR occurred in 48% (16/33) of contraindicated DDI. The contraindicated DDIs were associations of aspirin with warfarin or fluindione.

**TABLE 6:** Frequencies of contraindicated drug–drug interactions and their nature of risk.

Name of ward	INN of drugs implicated in the contraindicated DDI		Number of contraindicated DDIs (n)					Nature of risk of the contraindicated DDI	Biological impact of contraindicated DDIs	
	Drug A	Drug B	Newborns	Infants	Children	Adolescents	All age groups combined		Type	Number of DDIs concerned
	**Total number of contraindicated DDIs**		**19**	**114**	**347**	**229**	**709**			
**Cardiology ward**	**Number of contraindicated DDIs in this hospital department:**		**19**	**82**	**167**	**29**	**297**			
	Potassium canrenoate + trometamol	potassium (CHL®)	13	38	1		52	Risk of hyperkalaemia	Hyperkalaemia	12
	Spironolactone	Potassium (Glucidion®)		3	31	2	36	Risk of hyperkalaemia	Hyperkalaemia	15
	Spironolactone	Potassium (CHL®)	2	21	10		33	Risk of hyperkalaemia	Hyperkalaemia	3
	Lysine acetylsalicylate	Fluindione		14	17		31	Increased risk of bleeding	INR value > INR target + SAD^a^ ‘Acquired coagulation factor deficiency’ and ‘Gastrointestinal bleeding’ + fall in factor VII + X to 10%, factor II to 19% → possible vitamin K‐dependent deficiency	6
	Potassium canrenoate + trometamol	Potassium (Numetah®)			25	1	26	Risk of hyperkalaemia	Hyperkalaemia	9
	Potassium canrenoate + trometamol	Potassium (Glucidion®)	1	1	18		20	Risk of hyperkalaemia	Hyperkalaemia	5
	Hydroxyzine	Amiodarone			18		18	Increased risk of ventricular arrhythmias		
	DOMPERIDONE	ESCITALOPRAM				13	13	Increased risk of ventricular arrhythmias		
	Spironolactone	Potassium (Numetah®)			10		10	Risk of hyperkalaemia		
	Nalbuphine	Morphine			8	1	9	Risk of the appearance of a withdrawal syndrome		
	Spironolactone	Potassium canrenoate + trometamol	2	2	4	1	9	Risk of hyperkalaemia	Hyperkalaemia	1
	Methadone	Hydroxyzine			8		8	Increased risk of ventricular arrhythmias		
	Spironolactone	Potassium (Diffu‐K®)			6	1	7	Risk of hyperkalaemia	Hyperkalaemia	1
	Gentamicin	Gentamicin			1	3	4	Increased risk of nephrotoxicity and ototoxicity	Hypercreatinine	1
	Levomepromazine	Hydroxyzine			4		4	Increased risk of ventricular arrhythmias		
	Spironolactone	Potassium (GLUCO®)		3	1		4	Risk of hyperkalaemia		
	Erythromycine	Escitalopram				3	3	Increased risk of ventricular arrhythmias		
	Hydroxyzine	Hydroxyzine			2	1	3	Increased risk of ventricular arrhythmias		
	Spironolactone	Potassium (RCA®)			2		2	Risk of hyperkalaemia	Hyperkalaemia	1
	Amikacine	Gentamicin				1	1	Increased risk of nephrotoxicity and ototoxicity		
	Cyamemazine	Hydroxyzine			1		1	Increased risk of ventricular arrhythmias		
	Domperidone	Erythromycin				1	1	Increased risk of ventricular arrhythmias		
	Hydroxyzine	Sotalol				1	1	Increased risk of ventricular arrhythmias		
	Nalbuphine	Sufentanil	1				1	Risk of the appearance of a withdrawal syndrome		
**Developmental psychopathology ward**	**Number of contraindicated DDIs in this hospital department:**				**20**	**101**	**121**			
	Cyamemazine	Hydroxyzine			13	72	85	Increased risk of ventricular arrhythmias		
	Cyamemazine	Escitalopram			5	14	19	Increased risk of ventricular arrhythmias		
	Methylphenidate	Methylphenidate				6	6	Risk of vasoconstriction and/or hypertensive crises		
	Cyamemazine	Mequitazine				5	5	Increased risk of ventricular arrhythmias		
	Cyamemazine	Domperidone				2	2	Increased risk of ventricular arrhythmias		
	Pimozide	Hydroxyzine			2		2	Increased risk of ventricular arrhythmias		
	Hydroxyzine	Escitalopram				1	1	Increased risk of ventricular arrhythmias		
	Hydroxyzine	Mequitazine				1	1	Increased risk of ventricular arrhythmias		
**Pulmonology, allergology and cystic fibrosis ward**	**Number of contraindicated DDIs in this hospital department:**				**77**	**15**	**92**			
	Tobramycine sulfate	Tobramycine sulfate			66	2	68	Increased risk of nephrotoxicity and ototoxicity		
	Amikacine	Amikacine			4	2	6	Increased risk of nephrotoxicity and ototoxicity	Hypercreatinine	4
	Domperidone	Moxifloxacine				5	5	Increased risk of ventricular arrhythmias		
	Cyamemazine	Hydroxyzine				4	4	Increased risk of ventricular arrhythmias	1 ADR: QT prolongation	1
	Amikacine	Tobramycine base			2		2	Increased risk of nephrotoxicity and ototoxicity		
	Spironolactone	Potassium (Transipeg®)			2		2	Risk of hyperkalaemia		
	Tobramycine sulfate	Tobramycine base			1	1	2	Increased risk of nephrotoxicity and ototoxicity	Hypercreatinine	1
	Cyamemazine	Hydroxyzine			1		1	Increased risk of ventricular arrhythmias		
	Gentamicin	Gentamicin				1	1	Increased risk of nephrotoxicity and ototoxicity		
	Hydroxyzine	Hydroxyzine			1		1	Increased risk of ventricular arrhythmias		
**Nephrology and rheumatology ward**	**Number of contraindicated DDIs in this hospital department:**			**16**	**19**	**4**	**39**			
	Spironolactone	Potassium (GLUCO®)		14			14	Risk of hyperkalaemia	Hyperkalaemia	3
	Amikacine	Amikacine		1	2	4	7	Increased risk of nephrotoxicity and ototoxicity	Hypercreatinine	4
	Gentamicin	Gentamicin			6		6	Increased risk of nephrotoxicity and ototoxicity	Hypercreatinine	2
	Alfuzosin	Lopinavir + ritonavir			3		3	Risk of increased adverse reactions of alfuzosin including hypotension		
	Cyamemazine	Hydroxyzine			3		3	Increased risk of ventricular arrhythmias		
	Lysine acetylsalicylate	Warfarin			2		2	Increased risk of bleeding	INR value > INR target	2
	Hydroxyzine	Hydroxyzine			2		2	Increased risk of ventricular arrhythmias		
	Acitretin	Concentrat synthetic vitamin A (Uvesterol®)		1			1	Risk of symptoms suggestive of hypervitaminosis A		
	Levomepromazine	Hydroxyzine			1		1	Increased risk of ventricular arrhythmias		
**Hepato‐gastrology ward**	**Number of contraindicated DDIs in this hospital department:**			**10**	**26**	**2**	**38**			
	Cyamemazine	Erythromycin			14		14	Increased risk of ventricular arrhythmias		
	Potassium canrenoate + trometamol	Potassium (CHL®)		6			6	Risk of hyperkalaemia		
	Spironolactone	Potassium (GLUCIDION®)			6		6	Risk of hyperkalaemia		
	Gentamicin	Gentamicin		1	2		3	Increased risk of nephrotoxicity and ototoxicity		
	Digoxin	Potassium (POLYIONIQUE B®)		2			2	Risk of serious or even fatal heart rhythm disorders		
	Hydroxyzine	Hydroxyzine			2		2	Increased risk of ventricular arrhythmias		
	Amikacine	Gentamicin		1			1	Increased risk of nephrotoxicity and ototoxicity		
	Cyamemazine	Hydroxyzine			1		1	Increased risk of ventricular arrhythmias		
	Erythromycine	Hydroxyzine			1		1	Increased risk of ventricular arrhythmias		
	Hydroxyzine	Mequitazine				1	1	Increased risk of ventricular arrhythmias		
	Nalbuphine	Morphine				1	1	Risk of the appearance of a withdrawal syndrome		
**Endocrinology and diabetology ward**	**Number of contraindicated DDIs in this hospital department:**			**1**	**4**	**6**	**11**			
	Cyamemazine	Mequitazine				6	6	Increased risk of ventricular arrhythmias		
	Gentamicin	Gentamicin		1	2		3	Increased risk of nephrotoxicity and ototoxicity	Hypercreatinine	1
	Hydroxyzine	Hydroxyzine			1		1	Increased risk of ventricular arrhythmias		
	Linezolid	Tramadol			1		1	Risk of developing serotonin syndrome		
**Orthopaedic, traumatological and plasty surgery ward**	**Number of contraindicated DDIs in this hospital department:**				**7**	**56**	**63**			
	Amikacine	Amikacine				46	46	Increased risk of nephrotoxicity and ototoxicity		
	Gentamicin	Gentamicin			6	1	7	Increased risk of nephrotoxicity and ototoxicity	Hypercreatinine	1
	Hydroxyzine	Hydroxyzine			1	3	4	Increased risk of ventricular arrhythmias		
	Linezolid	Tramadol				4	4	Risk of developing serotonin syndrome		
	Amikacine	Gentamicin				2	2	Increased risk of nephrotoxicity and ototoxicity		
**Neurology ward**	**Number of contraindicated DDIs in this hospital department:**				**4**	**13**	**17**			
	Cyamemazine	Amiodarone				7	7	Increased risk of ventricular arrhythmias		
	Cyamemazine	Hydroxyzine			2	4	6	Increased risk of ventricular arrhythmias		
	Gentamicin	Gentamicin			2		2	Increased risk of nephrotoxicity and ototoxicity		
	Amikacine	Amikacine				1	1	Increased risk of nephrotoxicity and ototoxicity		
	Hydroxyzine	Amiodarone				1	1	Increased risk of ventricular arrhythmias		
**Metabolic and hereditary diseases ward**	**Number of contraindicated DDIs in this hospital department:**			**5**	**2**	**3**	**10**			
	Methadone	Hydroxyzine		5			5	Increased risk of ventricular arrhythmias		
	Gentamicin	Gentamicin				3	3	Increased risk of nephrotoxicity and ototoxicity		
	Hydroxyzine	Hydroxyzine			1		1	Increased risk of ventricular arrhythmias		
	Spironolactone	Spironolactone			1		1	Risk of hyperkalaemia	Hyperkalaemia	1
**Urogenital, visceral and thoracic surgery ward**	**Number of contraindicated DDIs in this hospital department:**				**20**		**20**			
	Erythromycin	Hydroxyzine			9		9	Increased risk of ventricular arrhythmias		
	Spironolactone	Potassium (Gluco®)			5		5	Risk of hyperkalaemia		
	Gentamicin	Gentamicin			3		3	Increased risk of nephrotoxicity and ototoxicity		
	Spironolactone	Potassium (RCA®)			3		3	Risk of hyperkalaemia		
**Emergency ward**	**Number of contraindicated DDIs in this hospital department:**				**1**		**1**			
	Amikacine	Amikacine			1		1	Increased risk of nephrotoxicity and ototoxicity	Hypercreatinine	1

Abbreviations: DDI, drug–drug interaction; INN, International Nonproprietary Name; INR, international normalized ratio; SAD, significant associated diagnosis.

^a^ Significant associated diagnosis refers to a condition, symptom, or other reason for seeking healthcare that coexists with the primary diagnosis. It may represent an additional distinct health problem (another condition), a complication of the primary morbidity, or a complication related to the treatment of the primary morbidity.

For one patient who was prescribed lysine acetylsalicylate and fluindione and presented INR values above the target INR, several significant associated diagnoses were noticed: ‘Acquired coagulation factor deficiency’ and followed by ‘Gastrointestinal bleeding’ and in parallel a prothrombin rate <5% (normal values: 70–100), decrease in factor VII + X down to 10% (normal values: 60%–120%), decrease in factor II to 13% (normal values: 70%–109%). The significant associated diagnosis refers to a condition, symptom, or other reason for seeking care that coexists with the primary diagnosis. It may correspond to an additional, distinct health problem (another condition), a complication of the primary morbidity, or a complication resulting from the treatment of the primary morbidity.

The primary and associated diagnoses were obtained from the Programme de Médicalisation des Systèmes d'Information (PMSI), the French National Programme for Hospital Information Systems. This database collects and structures information on hospital stays in France and is used for hospital financing through Diagnosis Related Groups.

- A QT prolongation occurred in one patient with cyamemazine/hydroxyzine (1/101). The risk of this DDI is ventricular rhythm disorders, in particular ‘torsades de pointes’. Continuous electrocardiographic monitoring is recommended.

Due to missing data, it is possible that some ADRs were undetected (medical examinations not carried out or missing biological analyses).

## 4 DISCUSSION AND CONCLUSIONS

This study represents one of the largest investigations to date of DDIs in hospitalized paediatric patients, including more than 4500 children across 21 paediatric wards. A major strength of this work lies not only in its size but also in its assessment of the real‐world biological and clinical consequences of DDIs.

Among the 4543 patients hospitalized between September 2013 and September 2015, 12.5% experienced at least one DDI, while 4.3% had at least one contraindicated DDI. The observed frequency of DDIs was lower than that reported in previous studies, where prevalence estimates range from 42% to 75%.^10^, ^11^, ^12^, ^13^, ^37^, ^38^, ^39^, ^40^ This discrepancy can be explained by several factors.

First, substantial variability exists among DDI detection algorithms. Beyond differences in the lists of interacting drug pairs, marked heterogeneity has been reported in the description of clinical effects, severity grading, mechanisms, and management recommendations across drug information resources.^41^ The use of the Thériaque algorithm for DDI detection is, to our knowledge, unprecedented in paediatric inpatient studies. While this represents an innovative aspect of the present work, it also limits direct comparability with studies using international databases. Future research applying multiple DDI detection tools in parallel would help clarify these discrepancies.

Second, the study design contributed to the lower observed prevalence. Only DDIs classified as contraindicated or ‘not recommended’ were considered, whereas many published studies include all severity levels (minor, moderate, major, and contraindicated). As previously highlighted, there is no international consensus regarding DDI severity classification or terminology, which further complicates cross‐study comparisons.^42^

Third, characteristics of the study population likely influenced the findings. Patients were recruited from 21 paediatric departments and did not specifically include intensive care or emergency units, where disease severity and therapeutic complexity often necessitate aggressive pharmacological strategies. Patients with life‐threatening conditions typically receive multiple medications and are therefore at increased risk of DDIs.^43^ In addition, the presence of a computerized physician order entry system may have contributed to the lower rate of potential DDIs by alerting prescribers at the time of ordering.^37^

Most contraindicated DDIs (72%) were concentrated in only three departments. This distribution may reflect the single‐centre design of the study and local prescribing practices, which can differ substantially between countries and healthcare systems.^40^ The tertiary‐care context, with easier access to specialized care, also contrasts with studies conducted in resource‐limited environments.

A clear age‐related gradient was observed, with lower rates of contraindicated DDIs in newborns and infants compared with children and adolescents. Although newborns and infants represented more than half of the study population (50.8%), approximately 80% of contraindicated DDIs occurred in children aged 2–11 years and adolescents aged 12–15 years. This pattern likely reflects lower overall drug exposure in younger patients, whereas older children often receive more complex treatment regimens for chronic conditions. As children transition into adolescence, the risk of DDIs increasingly reflects to medication combinations rather than developmental pharmacokinetics alone.^44^ However, we were unable to demonstrate a direct relationship between prescription volume and DDI occurrence.

Several limitations related to the DDI detection algorithm should be acknowledged. Some DDIs were classified as contraindicated only under specific conditions that were not evaluated by the algorithm. For instance, none of the DDIs involving lysine acetylsalicylate and vitamin K antagonists were confirmed as contraindicated because aspirin doses were below the threshold associated with risk. Similarly, DDIs involving multiple formulations of aminoglycosides were often detected algorithmically but reflected prescribing practices rather than true pharmacological interactions. Although some cases of elevated serum creatinine were observed, these likely reflect the intrinsic nephrotoxicity of aminoglycosides rather than DDI‐specific effects.

Overall, our results suggest that contraindicated DDIs in hospitalized children rarely translate into clinically significant adverse events. Only one adverse drug reaction involving QT prolongation was observed and was classified as non‐serious, without prolongation of hospital stay. Moreover, even when biological abnormalities were detected, causal attribution to DDIs remained uncertain. For example, coagulation abnormalities and gastrointestinal bleeding observed in a patient receiving lysine acetylsalicylate and fluindione may be attributable to vitamin K antagonist overexposure alone, as aspirin does not influence coagulation factor synthesis or INR.

While these findings are reassuring, they should not be generalized beyond the hospital setting. Inpatient care benefits from prescription alert systems, close clinical supervision, and frequent biological monitoring, which likely mitigate the consequences of DDIs. These safeguards are less consistently available in outpatient care, where further studies are required to assess DDI‐related risks in paediatric populations.

The discrepancy between the frequency of contraindicated DDIs and the low incidence of adverse events raises important questions regarding the clinical relevance of DDI classifications and the effectiveness of alert systems. Notably, the classification of DDIs involving potassium‐sparing diuretics and potassium salts has been revised since the end of this study and is no longer considered contraindicated but ‘not recommended’ by French regulatory authorities.

Several initiatives have aimed to address inappropriate prescribing and DDIs in paediatrics, including tools designed to identify high‐risk prescriptions such as the POPI project,^45^ which aims to detect inappropriate drug prescriptions and target drugs whose combination could induce DDI. However, alert systems based solely on regulatory documentation may fail to detect relevant pharmacokinetic interactions that remain unpublished. Predictive tools capable of identifying CYP450‐mediated DDIs, including previously unreported interactions such as DDI‐predictor a free online tool,^46^ have shown promise in improving detection rates by hospital pharmacists.

More recently, artificial intelligence‐based approaches have emerged as potential solutions to improve DDI prediction, detection, and assessment of clinical consequences.^47^, ^48^, ^49^, ^50^ Such tools may ultimately enhance the clinical relevance of DDI alerts and reduce alert fatigue.

Although several studies assessing clinically significant DDIs in paediatric patients have been conducted outside Europe,^51^, ^52^, ^53^, ^54^ there is, to our knowledge, a lack of European inpatient paediatric studies simultaneously evaluating DDIs and associated adverse drug reactions.

Access to robust, up‐to‐date clinical decision support systems is essential for paediatric prescribers. Such systems could reduce inappropriate drug use, support dose optimisation, quantify the risk of adverse drug reactions associated with DDIs, and facilitate benefit–risk–based prescribing. Ultimately, this would improve therapeutic decision‐making in children with complex medical conditions and support the development of safer treatment strategies.

Clinical decision support systems (CDSSs) assist clinicians in applying best practices and integrating patient‐specific data to improve safety. A recent systematic review^55^ highlighted that, although numerous medication‐related CDSSs have been developed for neonatal and paediatric care, very few have been evaluated in real‐world clinical settings, with evidence largely limited to observational studies. Further research is therefore required to assess the implementation and effectiveness of medication administration CDSSs in routine practice.

In conclusion, although many DDIs were identified in hospitalized paediatric patients at Lyon Hospital, very few were associated with clinically significant consequences. However, these findings must be interpreted cautiously, given the use of an algorithm‐based detection approach, the lack of detailed pharmacokinetic and temporal data, and the restricted availability of clinical covariates. These methodological constraints may have influenced both the identification of DDIs and the assessment of their clinical consequences. Further studies using more granular exposure data and prospective outcome assessment are needed to better define the clinical relevance of DDIs in paediatric inpatient care and to inform the optimisation of decision‐support systems.

## AUTHOR CONTRIBUTIONS

**Emilie Laval**: Data curation; formal analysis; writing—original draft; writing—review and editing (equal). **Sylvain Goutelle**: Writing—review and editing (equal). **Inesse Boussaha**: Data curation. Xavier Dode: Software—development of DDI algorithm in Theriaque. **Yanis Mimouni**: Data curation; investigation. **Kim An Nguyen**: Conceptualization; funding acquisition; investigation; methodology; writing—review and editing (equal). **Behrouz Kassai**: Conceptualization; funding acquisition; investigation; methodology; writing—review and editing (equal).

## CONFLICT OF INTEREST STATEMENT

The authors declare no conflict of interest.

## Data Availability

The data supporting the findings of this study are available from the corresponding author upon reasonable request. Some data may not be shared due to privacy or ethical restrictions.
